# Environmental Gradients Shaping the Freshwater Bryophyte Communities of Croatia (Western Balkans)

**DOI:** 10.3390/plants11121542

**Published:** 2022-06-09

**Authors:** Anja Rimac, Antun Alegro, Vedran Šegota, Nina Vuković, Nikola Koletić

**Affiliations:** Division of Botany, Department of Biology, Faculty of Science, University of Zagreb, Marulićev trg 20/II, 10000 Zagreb, Croatia; anja.rimac@biol.pmf.hr (A.R.); vedran.segota@biol.pmf.hr (V.Š.); nina.vukovic@biol.pmf.hr (N.V.); nickoletic@gmail.com (N.K.)

**Keywords:** bryophytes, macrophytes, rheophytes, Mediterranean, karstic rivers, water chemistry, bioclimatic variables

## Abstract

A comprehensive field survey of 527 sites on 293 watercourses across Croatia revealed 76 sites (14.42%) in which bryophytes were the dominant part of the macrophyte vegetation. Using classification and ordination analyses, we obtained five community types segregated across the gradients of several climatic, physiographic and water chemistry parameters. The *Didymodon tophaceus*–*Apopellia endiviifolia* and the *Berula erecta-Cratoneuron filicinum* communities were mostly confined to the clean and basic karstic rivers of the Dinaric Ecoregion under the influence of the Mediterranean climate, with the *Didymodon tophaceus–Apopellia endiviifolia* community being a tufa-forming community associated with the seasonally dry watercourses of small catchment areas and cascades along the larger karstic rivers, while the *Berula erecta–Cratoneuton filicinum* community was mostly associated with rivers with larger catchment areas and permanent flow. On the other hand, the *Oxyrrhynchium hians–Chiloscyphus pallescens* community and the *Fissidens pusillus–Veronica beccabunga* community were associated with eutrophic water restricted to small rivers of the Pannonian Ecoregion under the influence of the temperate climate and flowing over silicate bedrock. The most represented and widespread in Croatia was the *Cinclidotus* community, displaying the widest ecological range in the study. It was mostly associated with the relatively clean karstic rivers of large catchment areas belonging to the Dinaric Ecoregion, with the majority of the sites under the influence of a temperate climate with higher precipitation during the warm period of the year. The geographical patterns of the freshwater bryophyte communities showed that the relatively clean, fast and cold karstic rivers belonging to the Dinaric Ecoregion provide habitats that harbour a greater diversity of bryophyte communities than the watercourses of the Pannonian Ecoregion, where bryophyte-dominated communities are restricted to a small number of small lowland and semi-montane rivers and predominantly occupy periodically flooded microhabitats such as river margins.

## 1. Introduction

Bryophytes are an important part of freshwater biodiversity, inhabiting a wide variety of aquatic and riparian habitats and ecological and hydrological niches associated with running and standing waters [1,2]. They are a dominant part of macrophyte vegetation in headwater and mountain streams, where they thrive in oligotrophic, clear, cold water with fast and usually torrential flow over very stable rocky substrates in a harsh environment unsuitable for the majority of other macrophytes [3,4]. Here, a wide variety of adaptations enables them to withstand mechanical stress from high water velocity and associated drag forces and shear stress [5]. The same features enable their complete dominance in waterfalls, where they develop the most luxuriant communities [6]. Namely, in such turbulent habitats, the thin boundary layer positively affects the amount of CO_2_ available for photosynthesis, as well as nutrients. On the other hand, in middle and lower river sections dominated by unstable substrates of smaller fractions, bryophytes are confined to larger rocks or periodically flooded river margins and are subject to intensive competition with vascular plants, leading to lower cover or complete absence of bryophytes [5,7]. Furthermore, bryophytes make a particularly significant component of vegetation in highly seasonal and intermittent Mediterranean rivers [8], primarily due to a set of diverse adaptations enabling desiccation tolerance during dry periods, as well as endurance for mechanical stress from strong flash flows. In such conditions of impermanent flows, freshwater bryophyte assemblages are characterized by a higher share of hygrophyte and drought-tolerant species [8,9], whereas rheophytes dominate the vegetation of streams and rivers with permanent flows [10]. However, truly aquatic bryophytes are rather rare [5], with some authors completely disputing this category, suggesting that all rheophylous species should be regarded as facultative aquatics since they have at least some degree of desiccation tolerance [4].

The diversity of these species is governed by the heterogeneity of different environmental factors, which determine their presence or absence, as well as the community structure. While the presence and cover of bryophytes in freshwater habitats are primarily determined by riverbed stability and substrate size [4,6,11], diverse environmental factors influence diversity and community structure. Hydrological, physiographic, geological and climatological factors, as well as water chemistry, have been recognized as the main groups of parameters influencing freshwater bryophyte communities [4,6,8,11,12,13]. Furthermore, these communities, as well as particular species, have been recognized as sensitive to changes in the land use of the catchment area and to related habitat degradation and water pollution [6,13]. Accordingly, representatives of the group, as well as community parameters such as total bryophyte cover, species richness and composition, have been recognized as good bioindicators of water quality and the hydromorphological degradation of aquatic habitats [13,14,15,16,17,18]. Therefore, freshwater bryophytes have been included as a part of the aquatic vegetation in the assessment of the ecological status of waterbodies conducted according to the Water Framework Directive (WFD) [19]. This has been especially important in headwater streams, where bryophytes are by far the dominant part of vegetation [3], or in highly seasonal rivers, where they are the only macrophyte representatives during the summer months when these rivers dry out [8].

Several models regarding the environmental gradients influencing freshwater bryophyte communities exist for different European regions, covering, for example, small mountain streams in Germany [4], lower mountain streams in the boreal zone [20] and the bryophyte communities of upland and lowland sites in English and Welsh rivers [11]. A comprehensive study of bryophyte communities in highly seasonal Mediterranean rivers from six countries provided an insight into their diversity and composition as well as a predictive model of their distribution in the Mediterranean area [8]. Furthermore, bryophyte assemblages were investigated in the Alpine and Apennine mountain streams of Italy [3], while in Southeast Europe, research into freshwater bryophyte communities is mostly limited to Bulgaria, where several studies investigating the ecology and bioindication potential of these communities were conducted [13,16,21,22]. However, further research is needed on this subject in this bryologically understudied European region [23,24] as a basis for future monitoring and protection.

The only research on bryophyte communities in Croatian watercourses so far conducted dates back to the middle of the 20th century, when the tufa waterfalls of the Krka River [25,26] were studied, followed by the waterfalls of the Plitvice Lakes system [27] and by the Una River [28,29]. During the 1960s, work was focused on the relationship between macrozoobenthos, bryophytes and algae in freshwater communities [30,31,32,33,34,35,36,37,38] and the end of this short but very fruitful period also marked the end of the research into the freshwater bryophytes in Croatia up to the present day. Croatian territory, a part of Southeast Europe, is divided into the Pannonian and the Dinaric Ecoregion, with the latter subdivided into the Continental and the Mediterranean Subecoregion. Since these regions reflect the climatological, geological and hydrological heterogeneity of Croatia, it can be expected that the geographical segregation of the bryophyte communities follows this division and that those communities would show some degree of affinity towards a particular region.

Given that systematic and comprehensive studies on freshwater bryophyte communities have never been conducted in Croatia and do not exist with respect to the Western Balkans, including the corresponding part of the Mediterranean, we aimed to (1) explore the distribution, diversity and species composition of freshwater bryophyte communities; (2) explore the inherent variability of particular ecoregions in terms of bryophyte communities; and (3) identify the environmental gradients that influence bryophyte communities. This will improve the knowledge on this subject on the European level and provide a basis for further monitoring and protection, including the mitigation of the negative impacts of climate change and associated changes in hydrological regimes, as well as of human-induced eutrophication and changes in both hydrological regimes and morphology of streams and rivers.

## 2. Results

Bryophytes were the dominant component of macrophyte vegetation in 76 sites out of the 527 (14.42%) surveyed sites on streams and rivers situated across the whole Croatian territory and encompassing the heterogeneity of Croatian watercourses in terms of the recent typology of the waterbodies developed for WFD implementation. The majority of the sites (61, to be precise) were situated in the Dinaric Ecoregion, accounting for 31.12% of a total 196 sites surveyed within the particular region. The Dinaric–Continental Subecoregion was the richest, with as many as 40.23% of sites with bryophyte communities out of a total 87 sites surveyed, while 23.85% out of 109 sites in the Dinaric–Mediterranean Subecoregion harboured macrophyte vegetation with bryophyte predominance. On the other hand, this proportion was comparatively low in the Pannonian Ecoregion, amounting to only 4.53%.

A total of 130 macrophyte taxa were recorded in bryophyte dominated sites, i.e., 68 bryophyte and 43 vascular plant species, along with 19 macroalgae taxa. Among 68 bryophyte species, 59 were mosses (Bryophyta) and only 9 were liverworts (Marchantiophyta) (Table S1). Overall mean bryophyte species richness was 7.57 ± 0.50 species per site. The most frequent bryophyte species, with a frequency of over 30%, were *Rhyncgostegium riparioides* (79.5%) and *Cratoneurn filicinum* (60.3%), followed by *Fontinalis antipyretica* (50.0%), *Cinclidotus fontinaloides* (44.9%), *Apopellia endiviifolia* (37.2%), *Cinclidotus aquaticus* (35.9%), *Fissidens crassipes* (35.9%) and, finally, *Cinclidotus riparius*, present in 34.6% of the 76 bryophyte-dominated sites. According to the classification proposed by Dierßen [10], the majority of the abovementioned species were rheophytes except for the amphyphyte *C. filicinum* and the hygrophyte *A. endiviifolia*. Regarding the vascular representatives, only 5 hydrophyte species were recorded, while the helophytes prevailed with as many as 38 species.

### 2.1. Community Groups

TWINSPAN classification of bryophyte-dominated sampling sites at maximal distance established five groups after three levels of division (Soerensen dissimilarity, max distance = 0.77). An ANOSIM test confirmed the overall significant difference among the TWINSPAN groups (coded hereafter as 1–5), i.e., the existence of discrete communities among the sampling sites (overall R = 0.50, *p*(same) < 0.0001) based on the species composition. Furthermore, ANOSIM pairwise comparisons showed that all community groups were significantly different (Table 1).

**Figure 1:**
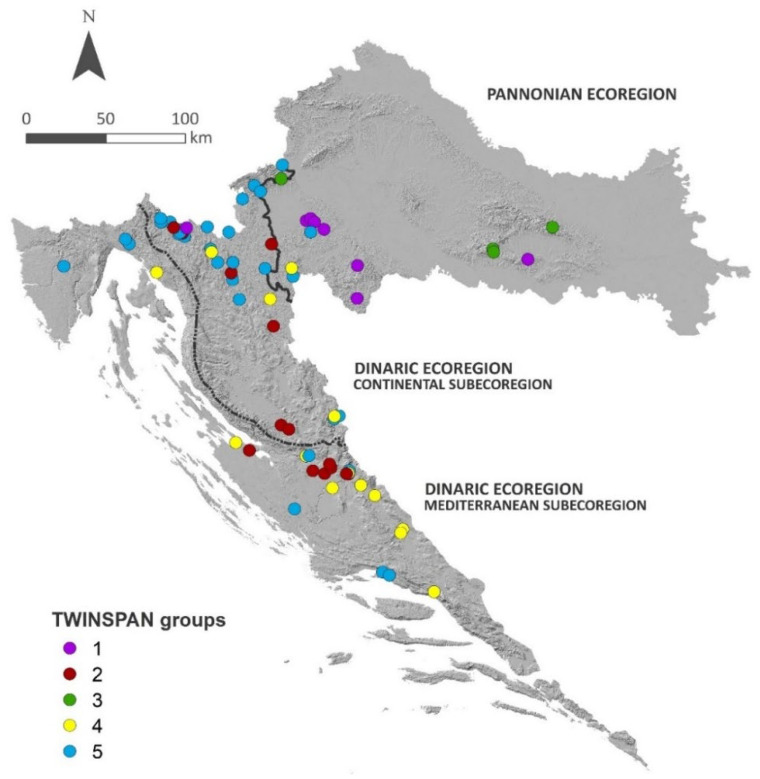
Distribution map of TWINSPAN groups in Croatia and its hydrological and biogeographical sub- and ecoregions.

With respect to the distribution of 76 bryophyte-dominated sites within the different sub- and ecoregions, 80.26% were located in the Dinaric Ecoregion (61 sites), with 46.05% (35 sites) situated in the Continental Subecoregion and 34.21% (26 sites) in the Mediterranean Subecoregion. The Pannonian Ecoregion was comparatively poor, with only 19.74% (15 sites) out of 76 sites (Figure 1, Table 2).

**Figure 2:**
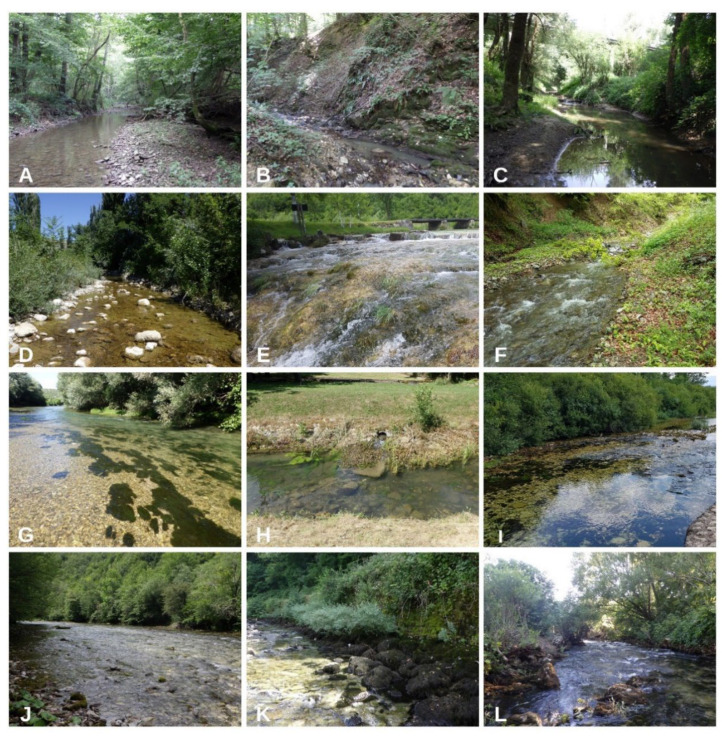
Examples of the sampling sites belonging to different TWINSPAN communities. Group 1: (**A**) Petrinjčica River (Miočinovići), (**B**) Vučjak River (Požega), (**C**) Kravarščica River (Dabići); Group 2: (**D**) Kobilica River (Kusac), (**E**) Korana River (settlement Korana); Group 3: (**F**) Šumetlica River (upper course); Group 4: (**G**) Krka River (Marasovine); (**H**) Joševica River (Donja Suvaja), (**I**) Cetina River (Barišići); Group 5: (**J**) Kupa (Kupari), (**K**) Rječina (Kukuljani), (**L**) Krupa (Mandići).

Some main patterns are recognisable when taking into account the distribution of particular TWINSPAN communities between the sub- and ecoregions; however, a certain overlap is present. Groups 1 and 3 are mainly confined to the Pannonian Ecoregion, while all others are more frequent in the Dinaric Ecoregion, with Group 2 being exclusive for this region and equally distributed in both the Continental and the Mediterranean subecoregions. On the other hand, Group 4 was more frequent in the Mediterranean Subecoregion, while Group 5 was more characteristic of the Continental Subecoregion (Figure 1 and Figure 2; Table 2).

**Figure 3:**
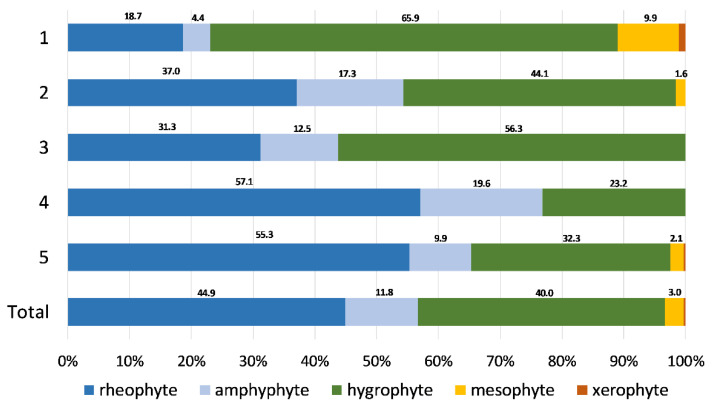
Bryophyte affinity to water for TWINSPAN groups and a total sample of 76 bryophyte-dominated sites.

The characteristic species of Group 1 (*Oxyrrhynchium hians–Chiloscyphus pallescens* community) (Appendix A) were mostly hygrophytic species confined to periodically submerged river margins, such as the mosses *Oxyrrhynchium hians*, *Plagiomnium undulatum, Pohlia melanodon*, and the liverworts *Chiloscyphus pallescens*, *Pellia neesiana*, *Conocephalum salebrosum*, along with the moss *Dichodontium pellucidum*, collected from periodically submerged rocks within the riverbeds. However, the constant species included rheophytes such as *Rhynchostegium riparioides* and *Leptodictyum riparium*, as well as an ampyphyte, *Cratoneuron filicinum*. The hygrophytes *Didymodon tophaceus*, *Apopellia endiviifolia* and *Funaria hygrometrica*, as well as the amphiphyte *Eucladium verticillatum* and the rheophyte *Fissidens crassipes*, were the characteristic species of Group 2 (*Didymodon tophaceus–Apopellia endiviifolia* community). Group 3 (*Fissidens pusillus–Veronica beccabunga* community) was characterized by bryophytes such as *Brachythecium rutabulum*, *Fissidens pusillus* and *Oxyrrhynchium speciosum*, as well as by the vascular helophytes *Veronica beccabunga* and *Persicaria dubia* (Appendix A). In general, Groups 1, 2 and 3 were characterized by a higher frequency of hygrophyte bryophyte species (1–65.9%, 2–44.1%, 3–56.3%), followed by rheophytes (1–18.7%, 2–37.0%, 3–31.3%) (Figure 3).

The characteristic species of Group 4 (*Berula erecta*–*Cratoneuron filicinum* community) were the vascular helophytes *Berula erecta*, *Menta aquatica* and *Sparganium erectum*, while the rheophyte mosses *Rhynchostegium riparioides* and *Fontinalis antipyretica* and the amphyphyte *Cratoneuron filicinum*, along with *Mentha aquatica*, were constant species with high frequencies within the group (Appendix A). This group was characterized by the highest frequency of rheophytes in all the groups (57.1%), followed by hygrophytes (23.2%) and amphyphytes (19.6%), while mesophyte and xerophyte bryophytes were completely absent (Figure 3). The characteristic species of Group 5 (*Cinclidotus* community) were *Cinclidotus riparius* and *Cinclidotus aquaticus*, both being constant species as well, along with *Cinclidotus fontinaloides*, *Fontinalis antipyretica*, *Rhynchostegium riparioides* and *Cratoneuron filicinum*. The rheophyte bryophytes were predominant in this group as well, accounting for 55.3% of all bryophyte occurrences within the group, followed by hygrophytes (32.3%) and amipyphytes (9.9%) (Figure 3).

Analysis of the TWINSPAN groups regarding macrophyte taxa richness revealed that sites belonging to the Group 1 had the highest mean value (12.25 ± 1.33), followed by Group 2 (10.56 ± 1.21) and Group 5 (10.24 ± 1.02). The same pattern was observed when considering the bryophyte species alone. Namely, the mean bryophyte species richness of sites belonging to Group 1 was highest (11.38 ± 1.18), again followed by Groups 2 (8.94 ± 0.91) and 5 (8.21 ± 0.83) (Table 3). The share of the bryophyte species in total number of taxa was the highest in Group 1 (86%) and over 50% within Groups 5 (56.3%) and 2 (59.3%), while in Group 4, it amounted to 33.33%. On the other hand, this group harboured the overall highest number of vascular plant species (28 species) and had a lower mean taxa richness and mean bryophyte species richness (4.4 ± 0.46) than Groups 1, 4, and 5. Finally, Group 3 was the most taxa-poor group, with the lowest mean taxa richness and mean bryophyte species richness (Table 3).

**Figure 4:**
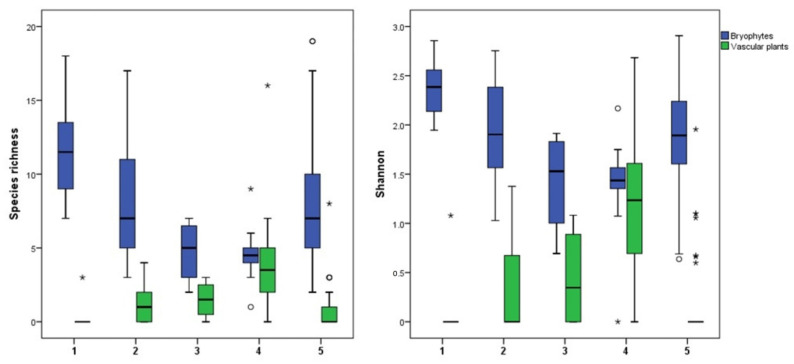
Comparison of bryophyte and vascular plant alpha diversity (species richness and Shannon-Wiener alpha diversity index) between the TWINSPAN groups (outliers: o—”out” values, *—”far out” or extreme values).

When present, vascular plants were mostly represented by helophyte species. A comparison of species richness and Shannon–Wiener alpha diversity index of bryophytes and vascular plants between the groups revealed that the vascular plant alpha diversity was the highest in Group 4, followed by Group 3, reaching that of bryophytes in some localities within Group 4 (Figure 4). By contrast, Groups 1 and 5 had a very low vascular plant alpha diversity; it was somewhat higher in Group 2, but still considerably lower than the bryophyte alpha diversity (Figure 4).

Mosses were the dominant representatives of bryophytes in all groups, representing over 80% of the total number of the bryophyte species in Groups 1 (83.8%), 2 (82.9%) and 5 (84.4%), and over 70% in Groups 3 (77.8%) and 4 (70.6%). Among the moss species, the plurocarpous prevailed in all groups.

**Figure 5:**
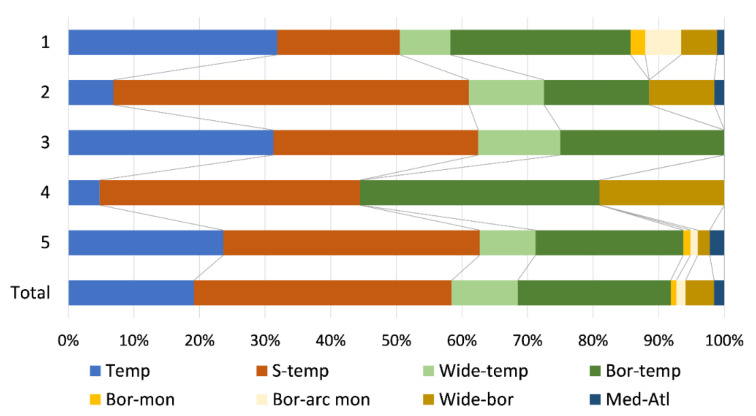
Chorological spectrum of freshwater bryophytes based on major biomes for TWINSPAN groups and a total sample of 76 bryophyte-dominated sites.

The chorological comparison of TWINSPAN groups based on major biomes revealed large chorotype overlapping, with a dominance of temperate chorotypes; however, some biogeographical differences were highlighted (Figure 5). The southern-temperate chorotype had the highest frequency in Group 2 (54.2%), while its lowest frequency was in Group 1 (18.7%). Furthermore, Group 4 was characterized by a higher frequency of boreo-temperate (36.5%) and wide-boreal elements (19.0%) than other groups. The Mediterranean–Atlantic chorotype was completely absent from Groups 3 and 4, with the highest, still quite low in proportion (2.2%) in Group 5. Boreal-montane and boreo-arctic montane chorotypes were most represented in Group 1, with 2.2% and 5.5% respectively.

The chorological comparison based on the eastern limit showed the dominance of circumpolar and European chorotypes in all communities, while other chorotypes were absent or present with frequencies lower than 5%, except for the Eurosiberian chorotype, which accounted for 7.7% of Group 5.

**Figure 6:**
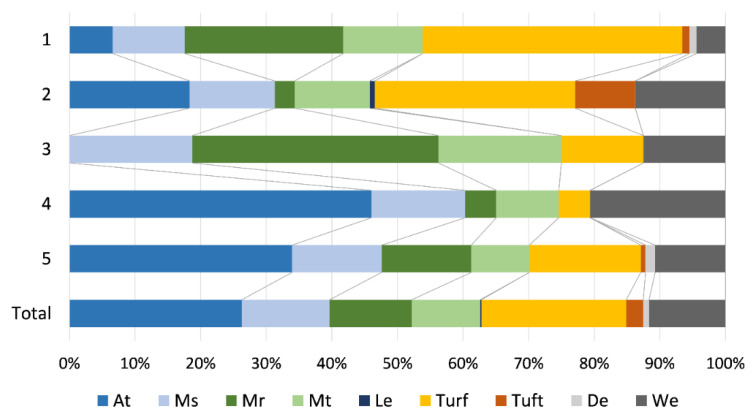
Lifeform spectrum of freshwater bryophytes for TWINSPAN groups and a total sample of 76 bryophyte-dominated sites (At—aquatic trailing, Ms—smooth mat, Mr—rough mat, Mt—thalloid mat, Le—lemnoid, De—dendroid, We—weft).

Bryophyte lifeforms were not evenly distributed among the TWINSPAN groups, with the most conspicuous difference in the share of aquatic trailings, rough mats and turfs (Figure 6). Namely, Groups 4 and 5 had the highest proportion of aquatic trailings, 46.0% and 33.9%, respectively, while this category was absent from Group 3 and represented with low frequency in Group 1 (6.6%). On the other hand, these latter two groups had a higher frequency of rough mats than the other groups, 37.5% for Group 3 and 24.2% for Group 1. Furthermore, Group 1 was characterized by a high frequency of turfs (39.56%), similar to Group 2, where turfs accounted for 30.5%.

**Figure 7:**
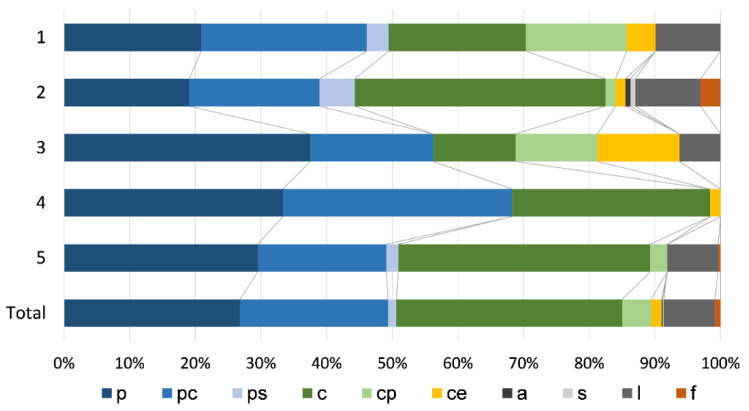
Life strategy spectrum of freshwater bryophytes for TWINSPAN groups and a total sample of 76 bryophyte dominated sites (p—perennials, pc—competitive perennials, ps—stress-tolerant perennials, c—colonist, cp—pioneer colonist, ce—ephemeral colonist, a—annual shuttle, s—short-lived shuttle, l—long-lived shuttle, f—fugitives).

Regarding the life strategies, all TWINSPAN groups feature the overall dominance of perennials, followed by colonist bryophyte species (Figure 7). The share of perennials was lowest in Group 2 (19.1%) and highest in Group 3 (37.5%), followed by Group 4 (33.3%). A similar pattern was revealed when observing all perennial categories together; they were most represented in Group 4 (68.3%), followed by Group 3 (56.3%) and Group 5 (50.9%). The share of colonists within Groups 2, 4 and 5 was over 35% and was lowest within Group 3 (12.5%). Taking into account all three colonist categories, the highest proportion was recorded in Group 2 (41.2%), followed by Groups 5 (40.9%) and 1 (40.7%).

### 2.2. Environmental Gradients

**Figure 8:**
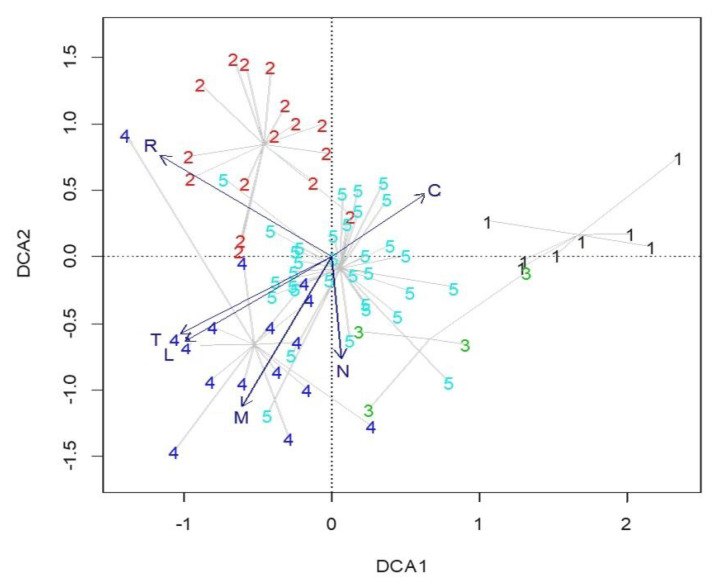
Ordination plot of DCA analysis with weighted Ellenberg indicator values passively projected as vectors over the ordination of TWINSPAN groups (C—continentality, L—light, M—moisture, N—nutrients, R—reaction, T—temperature).

DCA analysis showed the separation of discrete groups with some overlapping. The axis 1 eigenvalue was 0.34, and for axis 2, it was 0.29. The lengths of axes 1 and 2 were 3.8 and 2.9, respectively. The nature of the established gradients in the DCA analysis was further assessed with weighted Ellenberg indicator values, passively projected over the ordination as vectors. This revealed a gradient from the sites with higher mean indicator values for temperature, light and moisture and low values for continentality (Group 4) compared to those with higher values for continentality but lower temperature and light values (Group 1), with the sites belonging to Group 5 being intermediate across this gradient (Figure 8). Group 2 included the sites with higher Ellenberg indicator values for reaction. DCA axis 2 was the most strongly correlated with nutrient content, indicating the higher values in Group 3, as well as in some sites of Group 5.

**Figure 9:**
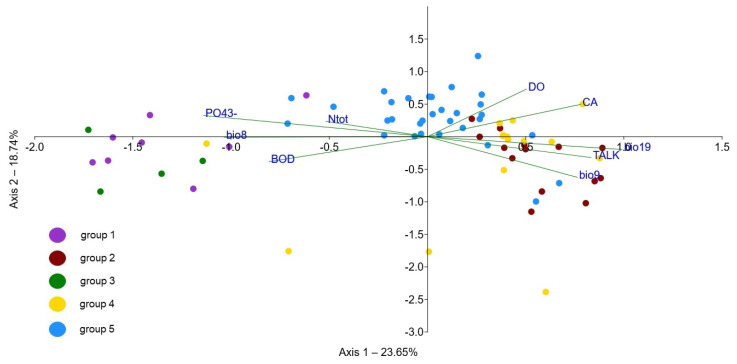
CCA ordination biplot with sampling sites belonging to different TWINSPAN groups and environmental variables (CA—catchment area, BOD—biochemical oxygen demand, DO—dissolved oxygen, N_tot_—total nitrogen, PO_4_^3−^—orthophosphates, TALK—total alkalinity, bio8—mean temperature of the wettest quarter, bio9—mean temperature of the driest quarter, bio19—precipitation of coldest quarter).

**Figure 10:**
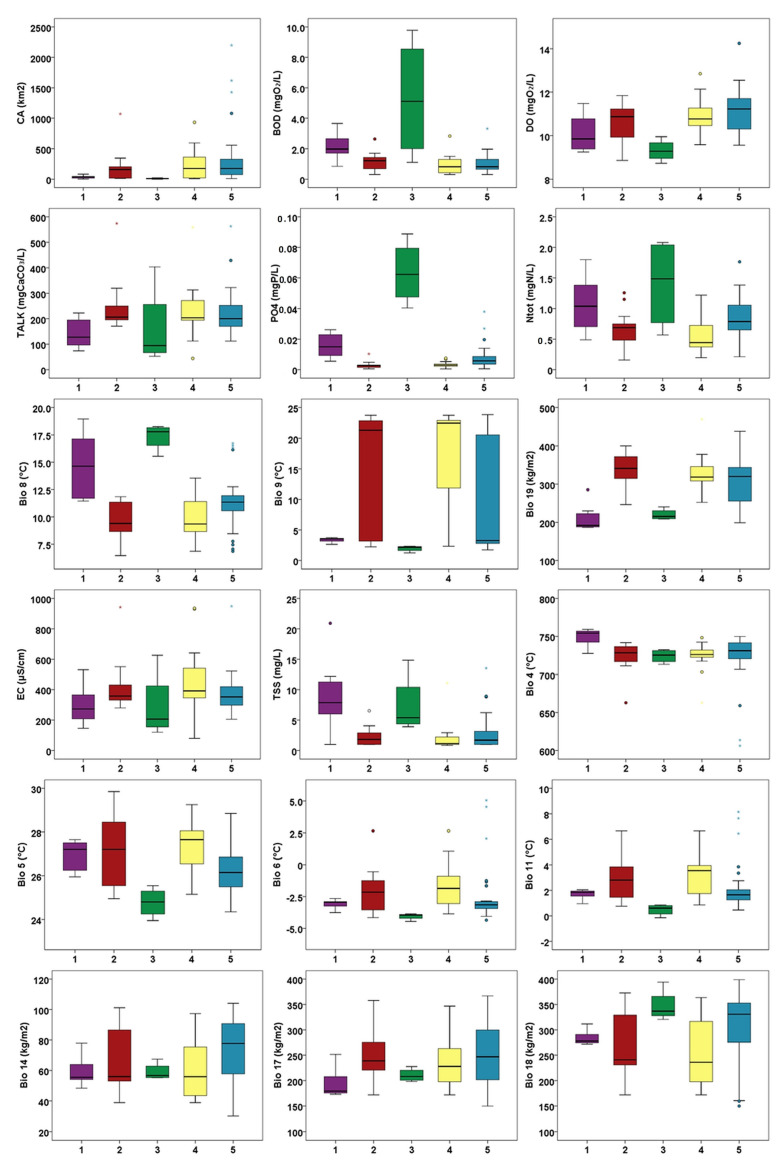
Boxplot diagrams of environmental variables included in CCA analysis and variables highly correlated (Spearman correlation coefficient ≥ |7|, *p* < 0.001) to CCA variables (for abbreviations, see Table 5; outliers: o—“out” values, *—“far out” or “extreme values”).

The first axis of the CCA explained 23.65% and the second axis explained 42.39% of the variation in the relationship between vegetation data and environmental factors. Eigenvalues of the first and second axes equalled 0.35 and 0.27, respectively. Ordination was statistically significant (F = 1.41, *p* = 0.001) according to the Monte Carlo permutation test (999 permutations). As expected, variables that explain the majority of the variance in the data were also highly correlated with axis 1. An ordination plot of the CCA analysis revealed a strong gradient along axis 1 from sites with high values of orthophosphates and biochemical oxygen demand, and elevated total nitrogen values (groups 1 and 3) to the sites with low values of these water chemistry parameters and high values of total alkalinity, as well as dissolved oxygen (Groups 2 and 4) (Table 4, Figure 9 and Figure 10). However, the Mann–Whitney pairwise test showed that the latter two groups differed significantly in pH values, with Group 2 being associated with more basic water (Appendix B and Appendix C). Sites belonging to Group 5 were distributed along the longest part of the CCA gradient, suggesting, in general, intermediate values for the abovementioned parameters compared to other groups. Total suspended solids (TSS) showed a similar pattern among the groups (Figure 10, Appendix B), and the Mann–Whitney pairwise test indicated a significant difference between Groups 1 and 3 with high values and the other three groups with low TSS (Appendix C).

Additionally, CCA indicated the importance of climatic variables in the segregation of the investigated sites along axis 1 as well. This was especially prominent for precipitation in the coldest quarter (bio19) with the highest values associated with sites belonging to Group 2, followed by Group 4. This variable was highly positively correlated with the precipitation of the wettest month (bio13, r_s_ = 0.97, *p* < 0.001) and the precipitation of the wettest quarter (bio16, r_s_ = 0.94, *p* < 0.001), the values of which had the same pattern among the groups. Similarly, Groups 2 and 4 were associated with higher values of the mean temperature of the driest quarter (bio9) than the other groups. This variable was highly and significantly (*p* < 0.001) correlated with other climatic variables (bio5, r_s_ = 0.89; bio6, r_s_ = 0.96; bio10, r_s_ = 0.91; bio11, r_s_ = 0.98; bio14, r_s_= −0.73; bio 18 r_s_= −0,86) describing the hot and dry summer conditions characteristic of the Mediterranean climate associated with the sites aggregated on the right side of the CCA plot, in contrast to the sites on the left end of the CCA axis 1, influenced by more temperate climatic conditions (Table 4). On the other hand, higher values of the mean temperature of the wettest quarter (bio8) were associated with the sites on the left part of the CCA ordination plot.

Regarding the physiographic variables, the catchment area was the most important in explaining the patterns in vegetation and environmental data, with groups 1 and 3 having generally smaller catchment areas (Table 4). This was corroborated by Mann–Whitney pairwise test; i.e., Group 3 was significantly different from Groups 2, 4 and 5, while Group 1 differed significantly from Groups 2 and 5 in the Mann–Whitney pairwise test (Appendix C).

## 3. Discussion

The present study is the first comprehensive study dealing with the freshwater bryophyte communities and the environmental gradients underpinning their diversity, composition and distribution in Croatia and the Western Balkans, filling the gap in the existing knowledge on this subject at the European level.

While the presence and cover of bryophytes in freshwater habitats are primarily determined by riverbed stability, substrate size, stream slope and localized flow type [4,6,11], the diversity and community structure are governed by environmental variables operating on a larger scale. Geological, physiographic, and climatic factors, as well as water chemistry parameters, have been identified as essential drivers shaping freshwater bryophyte communities [4,6,8,11,12,13,39].

Our findings confirm the importance of climatic, physiographic and water chemistry factors as major drivers influencing the diversity and composition of freshwater bryophyte communities, as well as their geographical segregation. Regarding water chemistry parameters, such as pH and alkalinity, which reflect the underlying geology, the distinction between hard- and soft-water bryoflora has been demonstrated by several authors on the European level and beyond [4,8,12,39,40,41,42]. Water trophy level, i.e., water nutrient content, has also been recognized as an important factor influencing bryophyte cover and diversity, as well as community structure, with anthropogenically influenced eutrophication having a detrimental impact on freshwater communities [13,14,15,16,18,41,43,44]. Finally, climatic factors, especially those related to precipitation and water availability, as well as their distribution over a year, have been proven to regulate freshwater bryophyte communities, especially in highly seasonal Mediterranean rivers [8,39].

In our study, communities confined to karstic rivers with high alkalinity and pH values, reflecting the dominant carbonate bedrock, and clean and oxygenated water (Groups 2 and 4), were characterized by basophilous species. The *Didymodon tophaceus–Apopellia endiviifolia* community (Group 2) showed a stronger bryophyte dominance in macrophyte species composition, with tufa-forming species such as *Didymodon tophaceus* and *Eucladium verticillatum* being among the characteristic species of the community, along with basophilous *Apopellia endiviifolia* and *Fissides crassipes*. The cooccurrence of other basophilus species of oligotrophic water, such as *Palustriella commutata* and *P. falcata* and the liverwort *Jungermannia atrovirens* [45], was recorded within the community as well. Similar species assemblages have already been described for calcareous rivers in Europe, especially from the Mediterranean area, and were regarded as typical of neutral to basic clean water with low nutrient content in undisturbed flush flow fed streams with regular or low current conditions, as well as from cascades [46,47,48]. This community showed a prevalence of hygrophyte taxa in our study, and this was corroborated by a high frequency of turfs, species with vertical stems with little or no branching [49], within the lifeform spectrum. These are known to thrive in seasonally flooded habitats, with the strong impact of water [50] suggesting an interplay between flash flows and low water table periods in the *Didymodon tophaceus–Apopellia endiviifolia* community. Additionally, this community had the highest proportion of colonists in the life-strategy spectrum, indicative of a higher share of microhabitats flooded seasonally with strong discharge [50]. Regarding bryophyte composition, the *Didymodon tophaceus–Apopellia endiviifolia* community corresponds well with the community described by Vieira et al. [8] as a freshwater bryophyte community that, in Mediterranean Europe, has an extensive predicted presence, according to environmental niche modelling, in highly seasonal rivers in Spain, southern France, Italy and Greece [8]. The same research concluded that this particular community was characteristic of highly seasonal streams at low to moderate altitudes and high values of precipitation in the driest quarter that sustain permanent flows. However, our study revealed that the most important bioclimatic variable influencing both this community and the *Berula erecta-Cratoneuron filicinum* community (Group 4) within Croatia was the precipitation of the coldest quarter, a good surrogate for the hydrological patterns of Mediterranean rivers, as well as the mean temperature of the driest quarter. These communities were associated with higher values for these bioclimatic parameters, characteristic of a Mediterranean climate with dry and hot summers where higher precipitation occurs during warm winters [51], and were subsequently characterized by the high proportion of southern-temperate chorotypes. On the other hand, they were characterized by overall large catchment areas, i.e., hydrological watersheds. Namely, Groups 2 and 4 were recorded on karstic rivers of the Dinaric Ecoregion, which are most often a part of large and complex hydrographic networks, and receive water from numerous springs, with overground and subterranean courses supplying water from the Dinaric mountains of both Croatia and neighbouring Bosnia and Herzegovina [52]. However, the *Didymodon tophaceus–Apopellia endiviifolia* community (Group 2) included several sites on intermittent rivers with small catchment areas, while on the sites with large catchment areas, it was mostly confined to cascades. The *Berula erecta-Cratoneuron filicinum* community (Group 4) had higher mean catchment area values and significantly lower water pH than the *Didymodon tophaceus–Apopellia endiviifolia* community. Being the transitional community in which vascular species start to outcompete bryophytes, it harboured the highest vascular species number and alpha diversity in comparison to all other groups. Vascular helophytes such as *Mentha aquatica*, *Berula erecta* and *Sparganium erectum* which thrive in the shallow and slower water along the river margins, indicated the gradual transition of completely bryophyte dominated vegetation towards the herbland vegetation of small freshwater streams and the shallow waterbodies of temperate Europe belonging to the alliance of *Glycerio–Sparganion* Br.-Bl. et Sissingh in Boer 1942 [53]. However, with the greatest proportion of rheophyte bryophytes confined to the riverbed, this was the most truly aquatic bryophyte community within the study. This was supported by the analysis of the lifeform spectrum, which revealed the highest proportion of aquatic trailings and a moderate proportion of smooth mats, lifeforms best adapted to permanent submergence [50]. Aquatic trailings are mostly associated with slower currents, whereas smooth mats prefer the more torrential water zones [2,50], so their ratio in the *Berula erecta–Cratoneuron filicinum* community reflects a permanent, slower and more streamlined flow. Regarding the life-strategy spectrum, perennial species were the most represented, indicating constant ecological and hydrological conditions in these watercourses.

The *Oxyrrhynchium hians–Chiloscyphus pallescens* community (Group 1) was mostly restricted to small lowland rivers located in the Pannonian Ecoregion [54], with a quite low mean value for the catchment area. The sites belonging to this community were characterized by eutrophic and turbid water with low alkalinity. The latter is a result of the underlying geology, since the silicate bedrock is dominant within the Pannonian Ecoregion, while the predominant substrates in these localities are sandy and gravelly alluvial deposits of silicate origin. Furthermore, the higher values of the mean temperature of the wettest quarter were associated with this group, indicating more temperate or even continental climatic conditions present in the Pannonian Ecoregion. The overall high proportion of hygrophytes within this community corresponds with the prevalence of turfs and rough mats (creeping pleorocarpous species with lateral branches erect) [49] in the lifeform spectrum, which inhabit periodically submerged margins of river stretches flowing through forests of the Pannonian lowland. Regarding the rheophytes, the most frequent were *Rhynchostegium riparioides* and *Laptodyctium riparium*. While *L. riparium* is unambiguously recognized as a pollution-tolerant aquatic moss [55], with a preference for eutrophic waters [16,17,41,44]*, R. riparioides* was omnipresent in our study, reaching the threshold set for constant species in all communities and being present along the entire gradient of nutrient concentration covered by this study, which suggests that the species is weakly linked to trophic conditions, highlighting its wide ecology range as previously reported by several authors [41,44].

The species-poorest community, *Fissidens pusillus–Veronica beccabunga* (Group 3), was the least represented in our study and mostly restricted to eutrophic and turbid small semi-montane watercourses within the Pannonian Ecoregion, with a single locality in the Dinaric–Continental Subecoregion on a small, lowland watercourse with a pebbly–gravelly substrate [54]. The vascular helophytes *Veronica beccabunga* and *Persicaria dubia* were among the constant and characteristic species of this community, which harboured the second highest vascular plant species richness following that of the *Berula erecta–Cratoneuron filicinum* community. The high share of hygrophytes, such as the characteristic species *Brachythecium rutabulum*, *Oxyrrhynchium speciosum* and *Marchantia polymorpha*, as well as a complete absence of aquatic trailings and a high share of rough mats (a lifeform with an adaptive advantage in microhabitats occurring above the normal level of maximum floods) [50] in the lifeform spectrum, confirmed that the periodically submerged rocks and alluvial sediments of river margins make the dominant microhabitat available to freshwater bryophytes in the watercourses of the Pannonian Ecoregion.

The *Cinclidotus* community (Group 5) was dominant in our study and displayed the widest ecological range regarding water quality and climatic variables associated with precipitation. While the majority of the sites were situated in the Dinaric–Continental Subecoregion, with a temperate climate with high values of precipitation in the warmest quarter, some of the sites were recorded in the source areas, as well as in the lower courses of Mediterranean rivers with permanent flow. Temperate chorotypes were dominant within the community, which was the case for all communities in the study, but here, they were represented by a high proportion of both southern-temperate and boreo-temperate elements. Additionally, the presence of the boreal, wide-boreal and Mediterranean–Atlantic chorotypes distinguished this community from the *Didymodon tophaceus–Apopellia endiviifolia* community and the *Berula erecta–Cratoneuron filicinum* community, which were dominantly under Mediterranean influence. Furthermore, the *Cinclidotus* community was characterized by neutral to basic water, related to the dominant carbonate bedrock in the area of its distribution and the slightly lower mean values of alkalinity in the *Didymodon tophaceus–Apopellia endiviifolia* and *Berula erecta–Cratoneuron filicinum* communities. The characteristic species of this group, *Cinclidotus aquaticus* and *C. riparius*, have already been reported in situations of high alkalinity and are associated with carbonate bedrock [39,56,57]. *Cinclidots aquaticus* is characteristic of clean, cold, well-oxygenated waters with low nutrient content [17,39] and is a typical species in the fast water of source areas, permanent torrential watercourses, rapids and waterfalls [39,58]. On the other hand, *C. riparius* was usually found in more sheltered microhabitats, not directly exposed to water dragging forces, while *C. fontinaloides*, a constant species of the *Cinclidotus* community, was most often found on periodically submerged rocks or tree stumps. Among constant species, *Fontinalis antypyretica* was the most truly aquatic species, with the least desiccation tolerance, growing completely submerged and attached either to rocks or to logs in moving water. The occurrence of *F. antipyretica* has not been closely related to specific physicochemical or trophic conditions in the majority of the available studies [11,14,20,44], although an increase in its frequency was observed with the decreasing concentrations of nitrates and phosphates [17]. Additionally, the constant species *Rhynchostegium riparioides* and *Cratoneuron filicinum*, as well as frequent cooccurrence of *Fissidens crassipess*, *Leptodyctium riparium* and *Apopellia endiviifolia*, make this community quite close to the bryophyte community most commonly found in the Mediterranean and predicted to occur in the freshwater streams of its eastern part, as well as in northern Spain and France [8]. This community was regarded as having a high proportion of exclusively aquatic species characteristic of riverbeds and many boreal elements as compared to other communities identified for the Mediterranean in the particular study. Similarly, the *Cinclidotus* community in our study was a prominently aquatic community with a high proportion of rheophyte species, which was corroborated by the high proportion of aquatic trailing, as well as smooth mats.

The geographical patterns of the freshwater bryophyte communities in Croatia show that the Dinaric Ecoregion provides substantially more suitable habitats than the Pannonian Ecoregion, harbouring all five communities identified in this study, with different bryophyte communities recorded at as many as 31.12% of all surveyed sites. This was expected, since fast, relatively clean and cold karstic rivers are a prominent feature of this ecoregion, and freshwater bryophytes are known to thrive and are the dominant component of the macrophyte vegetation in conditions of fast and turbulent flow, rocky substrates and low temperatures, which vascular plants cannot withstand [3,4,6,46,53,59]. They are a prominent part of the vegetation in highly seasonal Mediterranean rivers as well, where they successfully cope with the interchange of dry periods and periods with flash flows of strong water currents due to their diverse morphological and physiological adaptations. In contrast, the Pannonian Ecoregion harbours a very small number of sites with bryophyte vegetation, with only 4.53% of all surveyed sites having this vegetation type. Watercourses in the Pannonian Ecoregion are mostly slow, eutrophic lowland streams and rivers with unstable sandy and gravelly alluvial sediments unsuitable for bryophytes, which are here additionally subjected to competition with vascular plants [5,7]. Furthermore, the majority of these watercourses are subjected to flow regulation through canalization, riverbed deepening and embankment, as well as considerable changes in land-use practices, including the removal of the riparian vegetation [60], all of which have a negative impact on aquatic vegetation in general.

Our study was limited by the predefined survey sites that were included in the assessment of ecological status, which, according to the WFD, includes waterbodies with catchment areas greater than 10 km^2^, while omitting the majority of the source areas and smaller headwater streams in which bryophyte communities are expected to occur. With this in mind, future research should focus on these habitats, especially in parts of the Pannonian Ecoregion with mountain areas of high geological and geomorphological heterogeneity. However, we want to emphasise the importance of the WFD, which encouraged our research into freshwater bryophyte communities by including this group in its assessment of the ecological status of waterbodies. So far, substantial progress has been made [3,8,17,39], which is especially important in regions where bryophytes are still generally poorly researched [24], as in the case of Southeast Europe [13,16], with our findings contributing to knowledge with respect to the ecology and vegetation of bryophyte communities of the Mediterranean and providing the first insights into this subject for the Western Balkans.

## 4. Materials and Methods

### 4.1. Study Area

**Figure 11:**
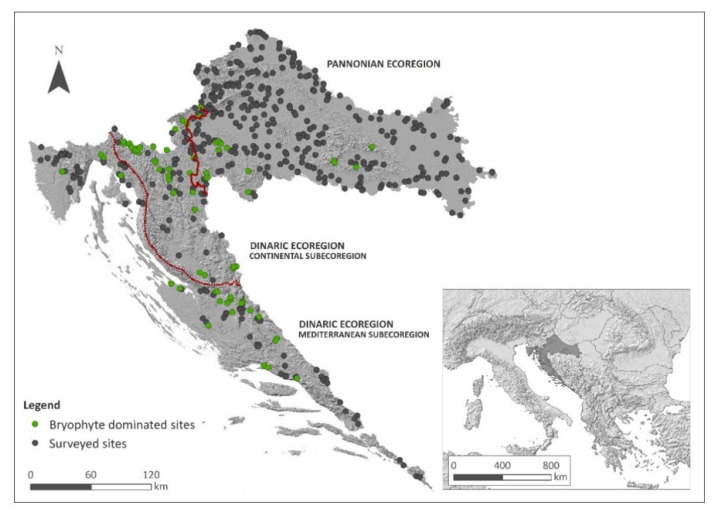
Study area with 527 sampling sites distributed across Croatia (Southeast Europe).

Data on the distribution of bryophyte-dominated freshwater communities was collected within the national surface water monitoring scheme conducted to assess the ecological status of waterbodies as required by the Water Framework Directive (WFD) [61]. The sampling sites were originally selected so as to encompass the heterogeneity of different waterbody types recognized by the recent typology developed as a basis for the monitoring of surface waters [54]. According to this typology, the land area of Croatia, 56,594 km^2^, is divided into two hydrological and biogeographical regions—the Pannonian and the Dinaric Ecoregion, the latter being subdivided into the Continental and Mediterranean Subecoregion (Figure 11). In all, 293 watercourses were surveyed during the vegetation seasons from 2016 to 2021. The survey included as many as 527 sampling sites, ultimately covering the whole of Croatian territory (Figure 11).

The Pannonian Ecoregion refers to the alluvial and diluvial plains in the inland part of the country bounded by three large rivers (Sava, Drava and Danube). This area ranges between 80 and 135 m a.s.l., with a small number of rather low, solitary mountain massifs reaching 1000 m a.s.l. The lithological and geological composition is mostly silicate quaternary deposits, while limestone is present only locally, in higher mountain areas. The climate is temperate, with warm summers throughout most of the area (Cfb), hot summers predominately in the eastern part (Cfa) and no dry season [62]. On the contrary, the Dinaric Ecoregion is predominately built from Mesozoic limestone and dolomite bedrock and is characterized by karstic phenomena. This ecoregion includes the Dinarides, the largest uninterrupted karst landscape in Europe, occupying almost 50% of the territory of Croatia. Because of the predominantly calcareous and dolomite bedrock, many rivers in the area have partly subterranean courses, or flow through impressive canyons or complex systems of barrage lakes, participating in the karst relief formation. The Continental Subecoregion is characterized by a temperate climate (Cfb), while the climate of the Mediterranean Subecoregion is mostly Mediterranean, i.e., temperate with dry and hot summer months (Csa) [62]. The Pannonian watercourses and the majority of the watercourses of the Dinaric–Continental Subecoregion belong to the Black Sea Basin, while the watercourses of the Dinaric–Mediterranean Subecoregion belong to the Adriatic Sea Basin.

### 4.2. Macrophyte Vegetation Sampling

A survey of macrophyte vegetation was performed according to the national methodology for macrophyte sampling [54] from June to September, when macrophyte vegetation is optimally developed, and during the lowest water discharge levels. Watercourses were surveyed for macrophytes along 100 m-long transects from the banks and, if the water depth was low enough, by zigzagging across the channel. In less accessible areas, the river bottom was raked to reach the macrophytes, with the rake either on a long pole or at the end of a rope.

Macrophyte survey included all representatives, i.e., bryophytes and vascular plants, as well as macroalgae. The survey included both vascular hydrophytes (truly aquatic macrophytes living submerged or floating on the water surface, rooted in the substrate or not) and helophytes (mostly marshland vascular species growing emergent along the water margins). Species coverage and abundance were assessed using the standard Central European methodology, i.e., extended nine-degree Braun–Blanquet scale (r = one individual; + = up to 5 individuals; 1 = up to 50 individuals; 2m = over 50 individuals, coverage < 5%; 2a = coverage 5–15%; 2b = coverage 15–25%; 3 = 25–50%; 4 = coverage 50–75%; 5 = coverage over 75%) [63,64,65].

Bryophytes were collected from various substrates (e.g., rocks, boulders, pebbles, xylal) within the riverbed, as well as from the periodically flooded river margins. Other macrophytes were collected for identification in the laboratory only if it was not possible to make accurate identification in the field. The collected material was deposited in herbarium ZA [66]. The nomenclature follows Hodgetts et. al. [67] for bryophytes, Euro + Med [68] for vascular plants and AlgaeBase [69] for algae.

### 4.3. Environmental Data Sampling and Collection

All localities were also sampled for basic water physicochemical and chemical parameters once a month throughout the year. Conductivity, pH, water temperature and dissolved oxygen were measured in situ with a Hach HQ40D Portable Multi Meter under standard conditions. Water samples were collected and analysed in an accredited laboratory (Central Water Management Laboratory, Zagreb, Croatia) for nine additional water chemistry parameters (Table 5).

Furthermore, physiographic and climatic environmental variables were obtained from several data sets using ArcGIS 10.5 software. Altitude was obtained from the digital elevation model EU-DEM v1.0 [70], distance from the source from topographic maps 1:25,000 [71], catchment area from the database of Hrvatske vode—the legal entity for water management and bioclimatic variables from CHELSA climatological datasets [72] (Table 5).

**Table 5:** Environmental variables and abbreviations used.

	Environmental Variable	Abbreviation
Water physicochemical parameters	Water temperature	T (°C)
	Water pH	pH
	Electrical conductivity	EC (μS/cm)
	Total suspended solids	TSS (mg/L)
	Dissolved oxygen	DO (mgO₂/L)
	Total alkalinity	TALK (mgCaCO₃/L)
	Biochemical oxygen demand	BOD (mgO₂/L)
Water chemical parameters	Ammonium	NH₄^+^ (mgN/L)
	Nitrites	NO_2_^−^ (mgN/L)
	Nitrates	NO_3_^−^ (mgN/L)
	Total nitrogen	N_tot_ (mgN/L)
	Orthophosphates	PO_4_^3−^ (mgP/L)
	Total phosphorus	P_tot_ (mgP/L)
Physiographical variables	Altitude	Alt (m a.s.l.)
	Catchment area	CA (km^2^)
	Distance from the source	DFS (m)
Climatic variables	Mean annual air temperature	Bio1 (°C)
	Mean diurnal air temperature range	Bio2 (°C)
	Isothermality	Bio3 (°C)
	Temperature seasonality	Bio4 (°C)
	Max temperature of the warmest month	Bio5 (°C)
	Min temperature of the coldest month	Bio6 (°C)
	Temperature annual range	Bio7 (°C)
	Mean temperature of wettest quarter	Bio8 (°C)
	Mean temperature of driest quarter	Bio9 (°C)
	Mean temperature of warmest quarter	Bio10 (°C)
	Mean temperature of coldest quarter	Bio11 (°C)
	Annual precipitation	Bio12 (kg/m^2^)
	Precipitation of wettest month	Bio13 (kg/m^2^)
	Precipitation of driest month	Bio14 (kg/m^2^)
	Precipitation seasonality	Bio15 (kg/m^2^)
	Precipitation of wettest quarter	Bio16 (kg/m^2^)
	Precipitation of driest quarter	Bio17 (kg/m^2^)
	Precipitation of warmest quarter	Bio18 (kg/m^2^)
	Precipitation of coldest quarter	Bio19 (kg/m^2^)

### 4.4. Data Analysis

Seventy-six sites in which bryophytes were the dominant component of macrophyte vegetation were selected for further analysis out of 527 surveyed sites. TWINSPAN analysis, a polythetic divisive classification method [73] modified by Roleček et al. [74], was conducted on vegetation relevés using Soerensen dissimilarity to assess whether discrete bryophyte communities occurred in any of the watercourses surveyed. TWINSPAN analysis was performed in Juice 7.1 [75]. The groups established by TWINSPAN were then tested for a significant difference based on species composition with the nonparametric test ANOSIM (using Bray–Curtis distance and 9999 permutations) [76] run in Past 4.9 software [77].

The groups resulting from these analyses were further analysed with respect to species composition. The synoptic table (Appendix A) was compiled in Juice 7.1. software. For all species within a particular group, e.g., community, φ-coefficients were calculated and tested for significance with the Fischer test. This statistical fidelity measure of particular species belonging to the previously defined groups was used to define characteristic species (φ ≥ 0.40, *p* < 0.05) [78]. Constant species were defined as those having the frequency within a particular group equal to or higher than 50%.

Bryophyte communities were analysed based on their affinity to water [10], chorotype composition [79], lifeform [49] and life strategies spectra [10] of bryophyte species. Furthermore, the alpha diversity (species richness and Simpson index) of the communities was analysed and visualized through boxplot graphs in SPSS 22.0 software.

Community structure was assessed using the indirect ordination method, Detrended correspondence analysis (DCA), run in R software (Vegan package) through Juice 7.1.-R connection. In DCA, weighted Ellenberg indicator values (EIV) for continentality, light, moisture, nutrients, temperature and pH reaction [49] were passively projected as vectors over the ordination to assess the possible environmental gradients. DCA revealed that the data were compositional with gradient longer than 3.0 SD units, indicating that a constrained analysis based on a unimodal model was most suitable for describing the data [80]. Subsequently, canonical correspondence analysis (CCA) was used as a direct ordination method to assess the relationship between environmental variables and patterns in species composition [81], while the statistical significance of the relationship between vegetation and environmental variables was tested by Monte Carlo permutation test using 999 permutations. Environmental data were log-transformed using the base-10 logarithm. Preselection of environmental variables was based on a correlation matrix between the variables and explorative DCA. Among highly positively correlated variables, those represented with the longest vectors in DCA were retained. This was performed in Past 4.9 software [77].

Additionally, a basic descriptive statistic (mean ± SE and min–max) of all environmental variables for groups derived from TWINSPAN analysis was calculated (Appendix B). A significant difference between groups was tested for each environmental variable with the nonparametric Kruskal–Wallace test, followed by Mann–Whitney pairwise *post hoc* tests (Appendix C). Nonparametric tests were selected since the majority of variables (32 out of 35) did not have a normal distribution, which was determined with the Shapiro–Wilk normality test in Past 4.9 [77].

## 5. Conclusions

The present study confirmed the importance of the climatic, physiographic and water chemistry gradients as major drivers shaping the diversity, composition and distribution of freshwater bryophyte communities. Comprehensive research revealed five community types and the patterns in their distribution across Croatia. Relatively clean and cold karstic rivers in the Dinaric Ecoregion that flow over carbonate bedrock and stable substrates represent far more suitable habitats, harbouring greater diversity of freshwater bryophyte communities in comparison to the rivers of the Pannonian Ecoregion. The *Didymodon tophaceus*–*Apopellia endiviifolia* and the *Berula erecta–Cratoneuton filicinum* are mostly confined to the karstic rivers of the Dinaric Ecoregion under the influence of the Mediterranean climate. The *Didymodon tophaceus–Apopellia endiviifolia* community is a tufa-forming community associated with the seasonally dry watercourses of small catchment areas and cascades along the larger karstic rivers, while the *Berula erecta–Cratoneuton filicinum* community is associated with rivers with a permanent and more streamlined flow. The *Cinclidotus* community is the most widespread in Croatia, having a wide ecological range and with the centre of its distribution being in the Dinaric–Continental Subecoregin, i.e., in fast and cold karstic rivers with permanent flow due to large catchment areas and high precipitation that are characteristic of this subecoregion. The species-rich *Oxyrrhynchium hians*–*Chiloscyphus pallescens* community and the species-poor *Fissidens pusillus*–*Veronica beccabunga* community are quite rare and are restricted to small eutrophic and turbid rivers of low alkalinity situated mainly in the Pannonian Ecoregion. These communities are characterized by a low share of rheophytes and a high share of species inhabiting the periodically flooded river margins, which is mainly related to low riverbed stability limiting the development of truly aquatic bryophyte communities in Pannonian rivers.

## Figures and Tables

**Table 1:** R statistics of the pairwise ANOSIMs of bryophyte-dominated communities obtained from TWINSPAN classification (groups 1–5) (overall R = 0.50, *p*(same) = 0.0001), * *p* < 0.001, ** *p* < 0.005.

	1	2	3	4	5
1					
2	0.68 *				
3	0.58 **	0.70 *			
4	0.66 *	0.36 *	0.56 **		
5	0.67 *	0.33 *	0.75 *	0.42 *	

**Table 2:** Distribution of the TWINSPAN groups over the hydrological and biogeographical sub- and ecoregions.

	Pannonian Ecoregion	Dinaric Ecoregion		Total
		Dinaric–Mediterranean Subecoregion	Dinaric–Continental Subecoregion	
Surveyed localities	331	109	87	527
Bryophyte-dominated localities	15	26	35	76
TWINSPAN group				
1	7	-	1	8
2	-	8	8	16
3	3	-	1	4
4	1	10	4	15
5	4	8	21	33

**Table 3:** Comparison of the number of taxa belonging to different macrophyte components and mean taxa and bryophyte species richness among TWINSPAN groups.

	TWINSPAN Groups					Total
	1	2	3	4	5	
Number of relevés	8	16	4	15	33	76
Number of taxa *	43	59	18	51	80	130
Bryophyte species	37	35	9	17	45	68
Mosses (Bryophyta)	31	29	7	12	38	59
pleurocarpous	14	11	6	7	18	25
acrocarpous	17	18	1	5	20	34
Liverworts	6	6	2	5	7	9
leafy	3	2	0	3	4	4
thallose	3	4	2	2	3	5
Vascular plant species	3	16	7	28	17	43
hydrophytes	0	0	0	1	5	5
helophytes	3	16	7	27	12	38
Macroalgae taxa *	3	8	2	6	18	19
Taxa richness						
mean ± SE	12.25 ± 1.33	10.56 ± 1.21	6.75 ± 1.49	9.27 ± 1.02	10.24 ± 1.02	10.14 ± 0.58
range (min–max)	7–18	4–24	3–10	4–20	4–25	3–25
Bryophyte species richness						
mean ± SE	11.38 ± 1.18	8.94 ± 0.91	4.00 ± 1.08	4.4 ± 0.46	8.21 ± 0.83	7.57 ± 0.50
range (min–max)	7–16	3–17	2–7	1–9	2–20	1–20

* Representatives of the genera *Batrachospermum* sp., *Mougeotia* sp., *Nostoc* sp., *Spirogyra* sp., *Vaucheria* sp. and *Zygnema* sp. were not identified at the species level.

**Table 4:** Summary of TWINSPAN groups’ main features.

**Group 1—*Oxyrrhynchium hians–Chiloscyphus pallescens* Community**
**Characteristic species:** *Oxyrrhynchium hians, Pellia neesiana, Conocephalum salebrosum, Fissidens taxifolius, Chiloscyphus pallescens, Plagiomnium undulatum, Dichodontium pellucidum, Pohlia melanodon, Hypnum cupressiforme, Plagiomnium ellipticum* **Constant species:** *Oxyrrhynchium hians, Pellia neesiana, Conocephalum salebrosum, Fissidens taxifolius, Chiloscyphus pallescens, Plagiomnium undulatum, Rhynchostegium riparioides, Cratoneuron filicinum, Ptychostomum pseudotriquetrum, Leptodyctium riparium* **Distribution:** Mainly Pannonian Ecoregion **Ecology:** Mostly restricted to the small lowland rivers with small catchment areas and under the influence of a temperate climate; in water with high values of orthophosphates, BOD and TSS, as well as low alkalinity due to silicate substrate; occurring on shaded habitats along river stretches flowing through forests. Characterized by high bryophyte richness, a high share of hygrophyte bryophytes growing on river margins and rough mats and turfs in lifeform spectrum.
**Group 2—*Didymodon tophaceus–Apopellia endiviifolia* Community**
**Characteristic species:** *Didymodon tophaceus, Eucladium verticillatum, Apopellia endiviifolia, Fissidens crassipes, Funaria hygrometrica* **Constant species:** *Didymodon tophaceus, Apopellia endiviifolia, Fissidens crassipes, Funaria hygrometrica, Rhynchostegium riparioides, Cratoneuron filicinum, Ptychostomum pseudotriquetrum, Cinclidotus fontinaloides* **Distribution:** Dinaric Ecoregion **Ecology:** Mainly tufa-forming community, occurring in karstic rivers with high alkalinity and pH values reflecting the dominant carbonate bedrock; in clean water with low nutrient content and BOD values and high dissolved oxygen levels. Characteristic for watercourses with considerable seasonality in water flow (intermittent rivers with small catchment areas, under the influence of the Mediterranean climate with dry and hot summers) and cascades in the lower courses of karstic rivers with larger catchment areas. Characterized by a high share of hygrophyte species and turfs in lifeform spectrum.
**Group 3—*Fissidens pusillus–Veronica beccabunga* Community**
**Characteristic species:** *Brachythecium rutabulum, Fissidens pusillus, Veronica beccabunga, Persicaria dubia, Oxyrrhynchium speciosum* **Constant species:** *Brachythecium rutabulum, Fissidens pusillus, Veronica beccabunga, Persicaria dubia, Oxyrrhynchium speciosum, Rhynchostegium riparioides, Cratoneuron filicinum, Marchantia polymorpha* **Distribution:** Mainly Pannonian Ecoregion **Ecology:** Occurring mainly in small, semi-montane watercourses with small catchment areas and under the influence of a temperate climate; in waters with high nutrient levels, BOD and TSS, and lower dissolved oxygen concentrations. Species-poor community, characterized by a higher share of hygrophytes and rough mats in lifeform spectrum, which grow on periodically submerged substrates.
**Group 4—*Berula erecta–Cratoneuron filicinum* Community**
**Characteristic species:** *Mentha aquatica, Berula erecta, Sparganium erectum* **Constant species:** *Cratoneuron filicinum, Rhynchostegium riparioides, Fontinalis antipyretica, Mentha aquatica* **Distribution:** Mainly Dinaric Ecoregion, Mediterranean Subecoregion **Ecology:** Transitional community of karstic rivers with large catchment areas and permanent flow, where vascular species start to outcompete bryophytes. Occurring in clean water with low nutrient content and BOD values, where helophytes occupy the river margins and shallower water, while bryophytes are confined to the riverbed. Characterized by a high share of rheophytes and aquatic trailings in lifeform spectrum, but low overall bryophyte species richness.
**Group 5—*Cinclidotus* Community**
**Characteristic species:** *Cinclidotus riparius, C. aquaticus* **Constant species:** *Cinclidotus riparius, C. aquaticus, C. fontinaloides, Rhynchostegium riparioides, Cratoneuron filicinum, Fontinalis antipyretica* **Distribution:** Mainly Dinaric Ecoregion, i.e., its Continental Subecoregion **Ecology:** The most widespread community, with a wide ecological range; in general, in waters with intermediate values of the water quality parameters (orthophosphates, total nitrogen and biochemical oxygen demand) and climatic variables associated with precipitation and water availability. Mostly in permanent karstic rivers with large catchment areas flowing over carbonate bedrock in neutral to basic water. Species-rich community characterized by a high share of rheophyte species and aquatic trailing in lifeform spectrum.

## Data Availability

The data presented in this study are available on request from the corresponding author.

## Supplementary Materials

The following supporting information can be downloaded at: https://www.mdpi.com/article/10.3390/plants11121542/s1, Table S1: Cover and abundance of the species at each surveyed locality with coordinates of localities in WGS84 (DIN-CON—Dinaric Ecoregion, Continental Subecoregion, DIN-MED—Dinaric Ecoregion, Continental Subecoregion, PAN—Pannonian Ecoregion).

## Appendix A. Synoptic Table with Percentage Frequency and Modified Fidelity Index φ-Coefficients

**Table A1:** Characteristic species (φ ≥ 40.00) are marked in bold, and constant species (frequency ≥ 50.00) are provided in grey cells.

TWINSPAN Group	1		2		3		4		5	
Number of Relevés	8		16		4		15		33	
*Oxyrrhynchium hians* (Hedw.) Loeske	**100**	**^94.6^**	.	^---^	.	^---^	.	^---^	9	^---^
*Pellia neesiana* (Gottsche) Limpr.	**50**	**^66.7^**	.	^---^	.	^---^	.	^---^	.	^---^
*Conocephalum salebrosum* Szweyk., Buczk. & Odrzyk.	**62**	**^63^**	.	^---^	.	^---^	.	^---^	18	^2.8^
*Fissidens taxifolius* Hedw.	**50**	**^61.3^**	6	^---^	.	^---^	.	^---^	.	^---^
*Chiloscyphus pallescens* (Ehrh.) Dumort.	**50**	**^58.7^**	.	^---^	.	^---^	7	^---^	3	^---^
*Plagiomnium undulatum* (Hedw.) T. J. Kop.	**50**	**^57^**	.	^---^	.	^---^	.	^---^	12	^---^
*Dichodontium pellucidum* (Hedw.) Schimp.	**38**	**^51^**	.	^---^	.	^---^	.	^---^	6	^---^
*Pohlia melanodon* (Brid.) A. J. Shaw	**38**	**^41.5^**	19	^---^	.	^---^	.	^---^	.	^---^
*Hypnum cupressiforme* Hedw.	**25**	**^45.9^**	.	^---^	.	^---^	.	^---^	.	^---^
*Plagiomnium ellipticum* (Brid.) T. J. Kop.	**25**	**^42.2^**		^---^	.	^---^	.	^---^	3	^---^
*Dichodontium flavescens* (Dicks.) Lindb.	25	^36.1^	.	^---^	.	^---^	.	^---^	9	^---^
*Didymodon tophaceus* (Brid.) Lisa	.	^---^	**50**	**^64^**	.	^---^	.	^---^	3	^---^
*Eucladium verticillatum* (With.) Bruch & Schimp.	.	^---^	**44**	**^59.1^**	.	^---^	.	^---^	3	^---^
*Apopellia endiviifolia* (Dicks.) Nebel & D. Quandt	.	^---^	**75**	**^49.6^**	.	^---^	40	^---^	33	^---^
*Fissidens crassipes* Wilson ex Bruch & Schimp.	12	^---^	**69**	**^48.8^**	.	^---^	.	^---^	48	^25.7^
*Funaria hygrometrica* Hedw.	.	^---^	**25**	**^42.2^**	.	^---^	.	^---^	3	^---^
*Rhynchostegium riparioides* (Hedw.) Cardot	75	^---^	81	^---^	75	^---^	60	^---^	94	^20.1^
*Cratoneuron filicinum* (Hedw.) Spruce	50	^---^	62	^---^	50	^---^	73	^---^	61	^---^
*Ptychostomum pseudotriquetrum* (Hedw.) J. R. Spence & H. P. Ramsay ex Holyoak & N. Pedersen	50	^---^	50	^31.8^	.	^---^	7	^---^	9	^---^
*Leptodictyum riparium* (Hedw.) Warnst.	50	^33.2^	.	^---^	.	^---^	13	^---^	48	^31.3^
*Cinclidotus fontinaloides* (Hedw.) P. Beauv.	.	^---^	56	^---^	.	^---^	33	^---^	64	^35.8^
*Fontinalis antipyretica* Hedw.	25	^---^	44	^---^	.	^---^	67	^---^	61	^---^
*Brachythecium rutabulum* (Hedw.) Schimp.	38	^24.1^	.	^---^	**50**	**^40.1^**	.	^---^	6	^---^
*Fissidens pusillus* (Wilson) Milde	38	^23.9^	.	^---^	**50**	**^40.0^**	7	^---^	.	^---^
*Veronica beccabunga* L.	.	^---^	.	^---^	**50**	**^66.7^**	.	^---^	.	^---^
*Persicaria dubia* (Stein) Fourr.	.	^---^	.	^---^	**50**	**^66.7^**	.	^---^	.	^---^
*Oxyrrhynchium speciosum* (Brid.) Warnst.	25	^---^	.	^---^	**50**	**^47.4^**	.	^---^	3	^---^
*Marchantia polymorpha* L.	12	^---^	12	^---^	50	^---^	7	^---^	33	^12.3^
*Hygroamblystegium tenax* (Hedw.) Jenn.	.	^---^	.	^---^	25		.	^---^	3	^---^
*Brachythecium rivulare* Schimp.	12	^---^	.	^---^	25	^---^	.	^---^	27	^21.3^
*Juncus buffonius* L.	.	^---^	.	^---^	25	^---^	.	^---^	.	^---^
*Mentha aquatica* L.	.	^---^	6	^---^	25	^---^	**60**	**^52.6^**	3	^---^
*Chiloscyphus polyanthos* (L.) Corda	38	^---^	6	^---^	.	^---^	47	^29.2^	21	^---^
*Berula erecta* (Huds.) Coville	.	^---^	6	^---^	.	^---^	**47**	**^52^**	9	^---^
*Sparganium erectum* L.	.	^---^	.	^---^	.	^---^	**27**	**^47.5^**	.	^---^
*Agrostis stolonifera L*.	.	^---^	25	^---^	25	^---^	33	^---^	15	^---^
*Vaucheria* A.P.de Candolle sp.	.	^---^	.	^---^	25	^---^	13	^---^	9	^---^
*Lythrum salicaria* L.	12	^---^	.	^---^	25	^---^	20	^---^	6	^---^
*Rorippa sylvestris* (L.) Besser	.	^---^	.	^---^	25	^---^	13	^---^	3	^---^
*Cinclidotus riparius* (Host ex Brid.) Arn.	12	^---^	25	^---^	.	^---^	7	^---^	**64**	**^51.2^**
*Cinclidotus aquaticus* (Hedw.) Bruch & Schimp.	.	^---^	19	^---^	.	^---^	40	^---^	**58**	**^40.6^**
*Cladophora glomerata* (Linnaeus) Kützing	.	^---^	19	^---^	25	^---^	13	^---^	33	^19.8^
*Hygroamblystegium varium* (Hedw.) Mönk.	25	^---^	6	^---^	.	^---^	.	^---^	6	^---^
*Heribaudiella fluviatilis* (Areschoug) Svedelius	25	^---^	.	^---^	.	^---^	7	^---^	6	^---^
*Mnium spinulosum* Bruch & Schimp.	12	^---^	.	^---^	.	^---^	.	^---^	.	^---^
*Fissidens bryoides* Hedw.	12	^---^	.	^---^	.	^---^	.	^---^	.	^---^
*Plagiomnium elatum* (Bruch et Schimp.) T. J. Kop.	12	^---^	.	^---^	.	^---^	.	^---^	.	^---^
*Brachythecium mildeanum* (Schimp.) Schimp	12	^---^	.	^---^	.	^---^	.	^---^	.	^---^
*Hygroamblystegium humile* (P.Beauv.) Vanderp., Goffinet & Hedenäs	12	^---^	.	^---^	.	^---^	.	^---^	.	^---^
*Bryum ruderale* Crundw. & Nyholm	12	^---^	.	^---^	.	^---^	.	^---^	.	^---^
*Leersia oryzoides* (L.) Sw.	12	^---^	.	^---^	.	^---^	.	^---^	.	^---^
*Scirpoides holoschoenus* (L.) Soják L.	.	^---^	19	^31.1^	.	^---^	7	^---^	.	^---^
*Batrachospermum* Roth sp.	.	^---^	19	^31.1^	.	^---^	7	^---^	.	^---^
*Calliergonella 24ectinate* (Hedw.) Loeske	.	^---^	19	^---^	.	^---^	.	^---^	9	^---^
*Spirogyra* Link. Sp.	.	^---^	19	^---^	.	^---^	.	^---^	9	^---^
*Jungermannia atrovirens* Dumort.	12	^---^	25	^---^	.	^---^	7	^---^	6	^---^
*Chara vulgaris* L.	.	^---^	19	^35.3^	.	^---^	.	^---^	3	^---^
*Dicranella varia* (Hedw.) Schimp.	.	^---^	12	^32^	.	^---^	.	^---^	.	^---^
*Palustriella 24ectinate* (Hedw.) Ochyra	.	^---^	12	^22.6^	.	^---^	7	^---^	.	^---^
*Palustriella falcata* (Brid.) Hedenäs	.	^---^	12	^9.3^	.	^---^	13	^10.8^	12	^8.6^
*Oenanthe fistulosa* L.	.	^---^	12	^---^	.	^---^	13	^---^	.	^---^
*Didymodon fallax* (Hedw.) R. H. Zander	.	^---^	12	^---^	.	^---^	.	^---^	15	^---^
*Deschampsia cespitosa* (L.) P. Beauv.	.	^---^	6	^---^	.	^---^	7	^---^	9	^---^
*Hygrohypnum luridum* (Hedw.) Jenn.	.	^---^	6	^---^	.	^---^	.	^---^	9	^---^
*Oxyrrhynchium schleicheri* (R. Hedw.) Röll	.	^---^	6	^---^	.	^---^	.	^---^	.	^---^
*Riccia fluitans* L.	.	^---^	6	^---^	.	^---^	.	^---^	.	^---^
*Veronica longifolia* L.	.	^---^	6	^---^	.	^---^	.	^---^	.	^---^
*Fissidens adianthoides* Hedw.	12	^---^	6	^---^	.	^---^	.	^---^	.	^---^
*Gymnostomum aeruginosum* Sm.	.	^---^	6	^---^	.	^---^	.	^---^	.	^---^
*Philonotis marchica* (Hedw.) Brid.	.	^---^	6	^---^	.	^---^	.	^---^	.	^---^
*Bryum dichotomum* Hedw.	.	^---^	6	^---^	.	^---^	.	^---^	.	^---^
*Alisma lanceolatum* With.	.	^---^	6	^---^	.	^---^	7	^---^	.	^---^
*Mentha longifolia* (L.) L.	.	^---^	6	^---^	.	^---^	7	^---^	.	^---^
*Iris pseudacorus* L.	.	^---^	6	^---^	.	^---^	7	^---^	.	^---^
*Bolboschoenus maritimus* (L.) Palla	.	^---^	6	^---^	.	^---^	.	^---^	.	^---^
*Juncus compressus* Jacq.	.	^---^	6	^---^	.	^---^	.	^---^	.	^---^
*Brachythecium salebrosum* (Hoffm. Ex F.Weber & D.Mohr) Schimp.	.	^---^	6	^---^	.	^---^	.	^---^	.	^---^
*Poa palustris* L.	.	^---^	6	^---^	.	^---^	.	^---^	.	^---^
*Fissidens arnoldii* R. Ruthe	.	^---^	6	^---^	.	^---^	.	^---^	.	^---^
*Trichostomum crispulum* Bruch	.	^---^	6	^---^	.	^---^	.	^---^	.	^---^
*Lemanea fluviatilis* (Linnaeus) C.Agardh	.	^---^	6	^---^	.	^---^	.	^---^	6	^---^
*Bangia atropurpurea* (Mertens ex Roth) C.Agardh	.	^---^	6	^---^	.	^---^	.	^---^	6	^---^
*Drepanocladus aduncus* (Hedw.) Warnst.	.	^---^	6	^---^	.	^---^	.	^---^	3	^---^
*Audouinella hermannii* (Roth) Duby	.	^---^	6	^---^	.	^---^	.	^---^	3	^---^
*Ranunculus trichophyllus* Chaix	.	^---^	.	^---^	.	^---^	20		6	^---^
*Lysimachia vulgaris* L.	.	^---^	.	^---^	.	^---^	13	^33.1^	.	^---^
*Lysimachia nummularia* L.	.	^---^	.	^---^	.	^---^	13	^33.1^	.	^---^
*Caltha palustris* L.	.	^---^	.	^---^	.	^---^	13	^33.1^	.	^---^
*Scirpus sylvaticus* L.	.	^---^	.	^---^	.	^---^	13	^33.1^	.	^---^
*Schoenoplectus lacustris* (L.) Palla	.	^---^	6	^---^	.	^---^	13	^---^	.	^---^
*Juncus 24ectinate24s* L.	.	^---^	6	^---^	.	^---^	13	^---^	6	^---^
*Nostoc* Vaucher ex Bornet & Flahault sp.	.	^---^	6	^---^	.	^---^	13	^---^	3	^---^
*Veronica anagallis-aquatica* L.	.	^---^	6	^---^	.	^---^	13	^---^	3	^---^
*Zygnema* C.Agardh sp.	12	^---^	.	^---^	.	^---^	13	^---^	3	^---^
*Lycopus europaeus* L.	.	^---^	.	^---^	.	^---^	13	^---^	3	^---^
*Hygroamblystegium fluviatile* (Hedw.) Loeske	.	^---^	.	^---^	.	^---^	7	^---^	.	^---^
*Alisma plantago-aquatica* L.	12	^---^	.	^---^	.	^---^	7	^---^	.	^---^
*Myosotis scorpioides* L.	.	^---^	.	^---^	.	^---^	7	^---^	.	^---^
*Samolus valerandi* L.	.	^---^	.	^---^	.	^---^	7	^---^	.	^---^
*Juncus inflexus* L.	.	^---^	.	^---^	.	^---^	7	^---^	.	^---^
*Ranunculus repens* L.	.	^---^	.	^---^	.	^---^	7	^---^	.	^---^
*Rorippa amphibia* (L.) Besser	.	^---^	.	^---^	.	^---^	7	^---^	.	^---^
*Helosciadium repens* (Jacq.) W. D. J. Koch	.	^---^	.	^---^	.	^---^	7	^---^	.	^---^
*Thamnobryum alopecurum* (Hedw.) Gangulee	12	^---^	.	^---^	.	^---^	.	^---^	12	^---^
*Fissidens fontanus* (Bach.Pyl.) Steud.	.	^---^	.	^---^	.	^---^	.	^---^	9	^---^
*Didymodon spadiceus* (Mitt.) Limpr.	.	^---^	.	^---^	.	^---^	.	^---^	9	^---^
*Rhynchostegiella teneriffae* (Mont.) Dirkse et Bouman	12	^---^	.	^---^	.	^---^	.	^---^	9	^---^
*Potamogeton nodosus* Poir.	.	^---^	.	^---^	.	^---^	.	^---^	9	^---^
*Didymodon luridus* Hornsch.	.	^---^	.	^---^	.	^---^	.	^---^	6	^---^
*Hildenbrandia rivularis* (Liebmann) J.Agardh	.	^---^	.	^---^	.	^---^	.	^---^	6	^---^
*Rhizoclonium hieroglyphicum* (C.Agardh) Kützing	12	^---^	.	^---^	.	^---^	.	^---^	6	^---^
*Mnium marginatum* (Dicks.) P.Beauv.	.	^---^	.	^---^	.	^---^	.	^---^	6	^---^
*Rhynchostegiella curviseta* (Brid.) Limpr.	.	^---^	.	^---^	.	^---^	.	^---^	3	^15.6^
*Lemanea rigida* (Sirodot) De Toni	.	^---^	.	^---^	.	^---^	.	^---^	3	^15.6^
*Potamogeton perfoliatus* L.	.	^---^	.	^---^	.	^---^	.	^---^	3	^---^
*Chaetophora lobata* Schrank	.	^---^	.	^---^	.	^---^	.	^---^	3	^---^
*Barbula unguiculata* Hedw.	12	^---^	.	^---^	.	^---^	.	^---^	3	^---^
*Stuckenia 25ectinate* (L.) Börner	.	^---^	.	^---^	.	^---^	.	^---^	3	^---^
*Carex acuta* L.	.	^---^	.	^---^	.	^---^	.	^---^	3	^---^
*Rhizomnium punctatum* (Hedw.) T.J.Kop.	12	^---^	.	^---^	.	^---^	.	^---^	3	^---^
*Tribonema viridae* Pascher	.	^---^	.	^---^	.	^---^	.	^---^	3	^---^
*Didymodon insulanus* (De Not.) M.O.Hill	.	^---^	.	^---^	.	^---^	.	^---^	3	^---^
*Myosoton aquaticum* (L.) Moench	.	^---^	.	^---^	.	^---^	.	^---^	3	^---^
*Fontinalis hypnoides* Hartm. Var. *duriaei* (Schimp.) Kindb.	.	^---^	.	^---^	.	^---^	.	^---^	3	^---^
*Mougeotia* C.Agardh sp.	.	^---^	.	^---^	.	^---^	.	^---^	3	^---^
*Galium palustre* L.	.	^---^	.	^---^	.	^---^	.	^---^	3	^---^
*Myriophyllum spicatum* L.	.	^---^	.	^---^	.	^---^	.	^---^	3	^---^
*Chara contraria* A.Braun ex Kützing	.	^---^	.	^---^	.	^---^	.	^---^	3	^---^
*Lophocolea bidentata* (L.) Dumort.	.	^---^	.	^---^	.	^---^	.	^---^	3	^---^
*Lemanea fucina* Bory	.	^---^	.	^---^	.	^---^	.	^---^	3	^---^

## Appendix B

**Table A2:** Basic descriptive statistic (mean ± SE and range (min–max)) of 35 environmental variables (for abbreviations, see Table 5).

TWINSPAN Group					
	1	2	3	4	5
ALT (m a.s.l.)					
mean ± SE	153.8 ± 20.6	282.3 ± 39.5	385.2 ± 56.2	231.1 ± 30.5	218 ± 18.7
min–max	102.3–227.9	93.0–564.0	290.1–538.8	31.4–376.6	0.3–384.2
DFS (km)					
mean ± SE	13.1 ± 2.5	19.9 ± 7.9	4.4 ± 0.8	6.6 ± 2.5	21.1 ± 5.1
min–max	1.4–21.5	3.6–116.0	3.2–6.5	0.2–33.8	0.04–118.2
CA (km^2^)					
mean ± SE	36.2 ± 8.5	198.5 ± 72.1	9.8 ± 4.2	230.7 ± 71.8	361.8 ± 93.2
min–max	2.7–79.8	13.3–1070.7	0.7–20.8	8.8–933.1	10.5–2199.4
T (°C)					
mean ± SE	12.3 ± 0.5	12.1 ± 0.6	14 ± 1.6	11.39 ± 0.45	12.01 ± 0.39
min–max	0.1–28.4	2.8–25.6	2.9–24.2	4.4–20.3	1.2–25.3
pH					
mean ± SE	7.70 ± 0.15	8.03 ± 0.05	8.08 ± 0.12	7.87 ± 0.04	7.99 ± 0.04
min–max	6.50–9.30	7.10–8.80	7.62–8.60	6.90–8.40	7.16–9.00
EC (μS/cm)					
mean ± SE	296.56 ± 43.34	416.29 ± 45.69	290.11 ± 114.12	459.71 ± 60.55	372.31 ± 24.36
min–max	69.00–722.00	129.00–1041.00	71.00–654.00	61.00–1049.00	8.00–1057.00
TSS (mg/L)					
mean ± SE	9.03 ± 2.07	2.19 ± 0.42	7.38 ± 2.52	2.12 ± 0.66	2.97 ± 0.53
min–max	<1.60–50.00	<0.53–17.2.	<1.60–33.00	<0.53–74.00	<0.53–69.00
TALK (mgCaCO₃/L)					
mean ± SE	141.91 ± 20.2	247.13 ± 27.69	161.19 ± 81.57	236.96 ± 29.35	213.24 ± 15.39
min–max	36.70–327.00	130.10–631.00	14.00–479.00	32.00–624.00	44.20–636.00
DO (mgO₂/L)					
mean ± SE	10.09 ± 0.29	10.63 ± 0.23	9.31 ± 0.25	10.88 ± 0.22	11.07 ± 0.15
min–max	5.46–14.55	6.95–14.00	2.72–13.16	7.50–14.01	4.90–16.90
BOD (mgO₂/L)					
mean ± SE	2.15 ± 0.30	1.17 ± 0.16	5.28 ± 1.99	0.97 ± 0.17	1.02 ± 0.10
min–max	<0.50–7.83	<0.09–4.60	0.81–16.47	<0.10–5.64	<0.10–14.3
NH₄^+^ (mgN/L)					
mean ± SE	0.126 ± 0.050	0.010 ± 0.003	0.0730 ± 0.0250	0.077 ± 0.057	0.026 ± 0.008
min–max	<0.003–<0.880	<0.0008–0.1480	<0.005–0.300	<0.003–3.420	<0.0008–1.277
NO_2_^−^ (mgN/L)					
mean ± SE	0.0215 ± 0.0135	0.0050 ± 0.0029	0.0189 ± 0.01411	0.0041 ± 0.0016	0.0063 ± 0.0018
min–max	0.0010–1.3030	0.0005–0.2210	0.0015–0.1100	0.0005–0.0790	0.0005–0.0900
NO_3_^−^ (mgN/L)					
mean ± SE	0.527 ± 0.114	0.427 ± 0.069	0.848 ± 0.224	0.424 ± 0.060	0.626 ± 0.053
min–max	0.001–2.615	0.017–1.871	0.070–2.180	0.010–1.400	0.010–2.391
N_tot_ (mgN/L)					
mean ± SE	1.067 ± 0.163	0.676 ± 0.080	1.405 ± 0.376	0.563 ± 0.072	0.847 ± 0.060
min–max	0.100–2.632	0.110–1.944	0.215–2.440	0.100–3.565	0.190–2.452
PO_4_^3−^ (mgP/L)					
mean ± SE	0.0157 ± 0.0029	0.0029 ± 0.0007	0.0635 ± 0.0104	0.0033 ± 0.0005	0.0079 ± 0.0014
min–max	<0.0040–<0.0900	<0.0012–0.0460	<0.0050–0.7600	<0.0010–0.0316	<0.0012–0.2020
P_tot_ (mgP/L)					
mean ± SE	0.0544 ± 0.0087	0.0122 ± 0.0028	0.1861 ± 0.043	0.0154 ± 0,0030	0.0232 ± 0.0200
min–max	<0.0030–<0.3200	<0.0015–0.1150	0.0200–0.7600	<0.002–0.1840	<0.002–0.3220
bio1 (°C)					
mean ± SE	11.58 ± 0.13	12.21 ± 0.42	9.95 ± 0.28	12.60 ± 0.36	11.67 ± 0.25
min–max	10.95–11.95	10.25–15.05	9.15–10.35	10.35–15.05	9.85–15.85
bio2 (°C)					
mean ± SE	9.18 ± 0.16	8.81 ± 0.25	8.93 ± 0.17	8.77 ± 0.29	8.38 ± 0.21
min–max	8.20–9.60	5.90–9.60	8.70–9.40	5.90–9.90	4.70–9.80
bio3 (°C)					
mean ± SE	30.53 ± 0.44	30.1 ± 0.56	31.03 ± 0.38	30.08 ± 0.63	29.06 ± 0.43
min–max	27.9–31.7	24.2–31.8	30.2–31.9	24.2–32.9	21.6–32.2
bio4 (°C)					
mean ± SE	749.30 ± 3.99	723.76 ± 5.48	724.23 ± 4.48	723.81 ± 5.11	722.45 ± 6.28
min–max	727.80–759.30	662.90–741.70	713.30–732.60	662.90–748.30	606.20–749.90
bio5 (°C)					
mean ± SE	26.94 ± 0.25	27.11 ± 0.45	24.78 ± 0.34	27.39 ± 0.31	26.22 ± 0.20
min–max	25.95–27.65	24.95–29.85	23.95–25.55	25.15–29.25	24.35–28.85
bio6 (°C)					
mean ± SE	−3.07 ± 0.13	-2.06 ± 0.5	−4.05 ± 0.14	−1.68 ± 0.46	−2.41 ± 0.41
min–max	−3.75–−2.65	−4.15–2.65	−4.45–−3.85	−3.85–2.65	−4.35–5.05
bio7 (°C)					
mean ± SE	30.01 ± 0.17	29.17 ± 0.38	28.83 ± 0.25	29.07 ± 0.40	28.63 ± 0.38
min–max	29.40–30.70	24.50–30.40	28.40–29.50	24.50–30.40	21.50–30.50
bio8 (°C)					
mean ± SE	14.68 ± 1.13	9.59 ± 0.47	17.35 ± 0.62	9.89 ± 0.47	11.36 ± 0.46
min–max	11.45–18.95	6.45–11.85	15.55–18.25	6.85–13.55	6.85–16.75
bio9 (°C)					
mean ± SE	3.38 ± 0.13	15.36 ± 2.62	1.98 ± 0.25	17.36 ± 2.34	8.50 ± 1.59
min–max	2.65–3.75	2.25–23.75	1.25–2.35	2.35–23.75	1.75–23.85
bio10 (°C)					
mean ± SE	21.13 ± 0.16	21.59 ± 0.43	19.23 ± 0.36	21.99 ± 0.35	20.93 ± 0.21
min–max	20.55–21.55	19.35–23.75	18.25–19.85	19.65–23.75	18.95–23.85
bio11 (°C)					
mean ± SE	1.71 ± 0.13	2.82 ± 0.47	0.48 ± 0.22	3.24 ± 0.42	2.21 ± 0.34
min–max	0.95–2.05	0.75–6.65	−0.15–0.85	0.85–6.65	0.45–8.15
bio12 (kg/m^2^)					
mean ± SE	1041.98 ± 41.05	1357.79 ± 49.47	1171.93 ± 52.28	1306.52 ± 55.65	1334.00 ± 43.27
min–max	960.80–1304.40	1090.00–1697.60	1101.30–1323.40	1077.80–1913.60	972.20–1801.90
bio13 (kg/m^2^)					
mean ± SE	110.90 ± 4.40	160.38 ± 5.07	133.43 ± 4.03	157.63 ± 6.79	151.32 ± 5.27
min–max	101.30–139.20	122.50–183.10	125.60–144.70	124.90–236.30	107.70–221.80
bio14 (kg/m^2^)					
mean ± SE	59.14 ± 3.29	65.30 ± 5.71	59.03 ± 2.87	61.23 ± 5.32	72.50 ± 3.73
min–max	48.30–78.00	38.90–101.20	55.30–67.40	38.90–97.30	30.20–104.10
bio15 (kg/m^2^)					
mean ± SE	18.54 ± 0.44	23.71 ± 1.41	21.05 ± 0.51	24.67 ± 1.61	21.87 ± 0.94
min–max	15.90–19.80	16.60–36.10	20.20–22.40	16.60–36.10	16.40–37.60
bio16 (kg/m^2^)					
mean ± SE	305.74 ± 14.9	441.85 ± 15.42	351.6 ± 18.67	430.73 ± 20.77	424.94 ± 15.27
min–max	279–405.7	339.9–524.4	323.8–406.6	343–675.3	299.1–619.7
bio17 (kg/m^2^)					
mean ± SE	193.66 ± 9.79	251.63 ± 14.65	210.55 ± 6.47	237.29 ± 13.50	251.38 ± 10.77
min–max	173.10–251.50	171.90–357.90	198.30–227.50	171.9–346.70	149.80–367.00
bio18 (kg/m^2^)					
mean ± SE	283.76 ± 4.78	267.39 ± 17.79	347.05 ± 16.07	251.72 ± 17.43	304.32 ± 12.03
min–max	272.00–311.80	171.90–372.80	320.80–393.90	171.90–363.70	149.80–398.80
bio19 (kg/m^2^)					
mean ± SE	209.61 ± 12.13	340.66 ± 11.69	219.98 ± 7.28	331.24 ± 12.60	304.98 ± 11.97
min–max	186.70–285.30	246.40–400.10	208.80–240.4	252.40–469.50	199.00–438.20

## Appendix C

**Table A3:** Results of Mann–Whitney pairwise post hoc tests; significant differences between TWINSPAN group pairs are marked with an asterisk (* *p* < 0.05, ** *p* < 0.001) (for abbreviations, see Table 5).

Environmental Variable	TWINSPAN Group Pairs Compared									
	1–2	1–3	1–4	1–5	2–3	2–4	2–5	3–4	3–5	4–5
T								*		
pH						*				
TSS	*		*	*	*			*	*	
TALK	*		*	*						
DO				*	*			*	*	
BOD	*		*	**	*			*	*	
NH_4_^+^	*			*	*			*	*	
NO_2_^−^	*		*		*			*		
NO_3_^−^										*
N_tot_			*					*		*
PO_4_^3-^	**	*	**	*	*		**	*	*	*
P_tot_	**	*	**	*	*		*	*	*	
ALT	*	*							*	
DFS			*		*	*				*
CA	*			*	*			*	*	
bio1		*			*			*	*	*
bio2				*						
bio3							*		*	
bio4	*	*	*	*						
bio5		*			*			*	*	*
bio6		*			*			*	*	*
bio7	*	*		*						
bio8	**		**	*	*			*	*	*
bio9		*	*		*			*	*	*
bio10		*			*			*	*	*
bio11		*			*			*	*	*
bio12	**	*	*	**						
bio13	**	*	**	**	*			*		
bio14				*						
bio15	*	*	*	*						
bio16	**	*	*	**	*			*		
bio17	*		*	*						
bio18		*								*
bio19	**		**	**	*			*	*	
